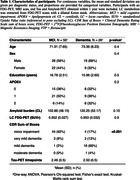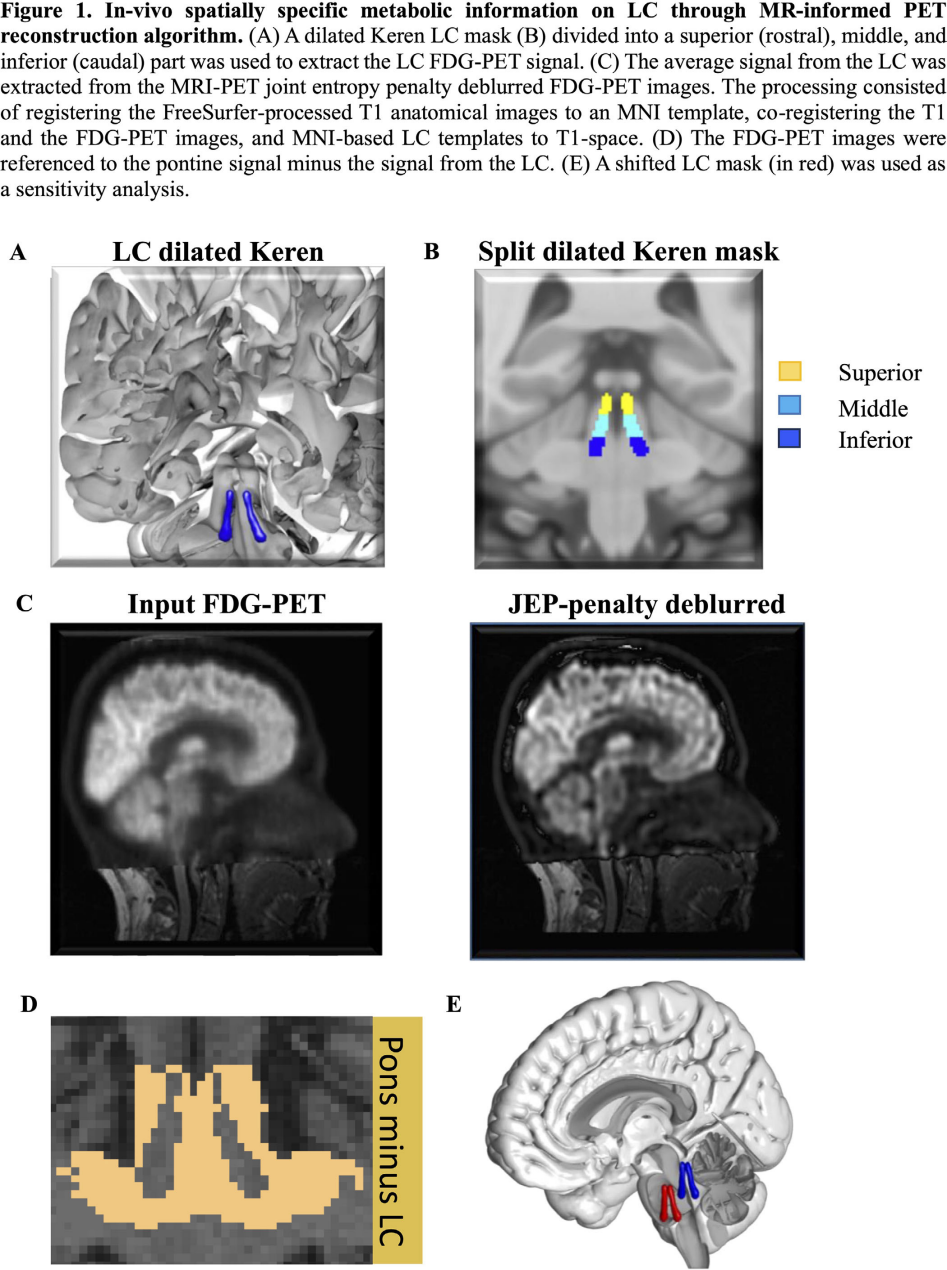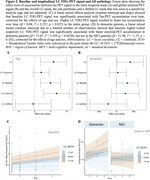# Locus coeruleus metabolism predicts the accumulation of Alzheimer’s disease pathology in amyloid‐positive symptomatic individuals

**DOI:** 10.1002/alz.094128

**Published:** 2025-01-09

**Authors:** Elouise A. Koops, Joyita Dutta, Alex Becker, Bernard J Hanseeuw, Reisa A Sperling, Keith A Johnson, Heidi I.L. Jacobs

**Affiliations:** ^1^ Gordon Center for Medical Imaging, Massachusetts General Hospital, Harvard Medical School, Boston, MA USA; ^2^ University of Massachusetts Amherst, Amherst, MA USA; ^3^ Center for Alzheimer Research and Treatment, Brigham and Women’s Hospital, Harvard Medical School, Boston, MA USA

## Abstract

**Background:**

Higher cerebrospinal fluid noradrenergic metabolic turnover has been associated with higher levels of Alzheimer’s disease pathology in cognitively impaired individuals. It remains unclear whether there is a specific anatomic vulnerability to metabolic alterations within the locus coeruleus (LC) and whether this hypermetabolism relates to steeper rates of pathology accumulation. Here, we overcome a spatial limitation of Positron Emission Tomography (PET) imaging in small nuclei with a dedicated Magnetic Resonance Imaging (MRI)‐guided framework to recover PET resolution. This method allowed us to investigate the [18F]Fluorodeoxyglucose (FDG)‐PET signal, reflecting energetic metabolic demand of the LC and its association with serial beta‐amyloid and tau‐PET.

**Methods:**

We included 78 amyloid‐positive, cognitively impaired participants from the ADNI‐3 cohort (Table 1). The LC was identified using a dilated Keren template registered to each T1 (Fig 1A). The LC mask was subdivided into a rostral, middle, and caudal part to investigate region‐specific associations (Fig 1B). We used an MR joint‐entropy‐penalty algorithm to quantify PET signal via deconvolution based on spatially‐variant blur kernels. The deconvolution step was stabilized using an anatomical‐based joint‐entropy prior (Fig 1C). The FDG‐PET signal was referenced to the pons, excluding the LC (Fig 1D). Amyloid positivity was determined as a centiloid score = 21, reflecting moderate neuritic plaques, on florbetaben and florbetapir scans. Linear mixed effects models investigated relationships between LC‐FDG, global beta‐amyloid and meta‐temporal tau burden (flortaucipir) over time. Age and sex were included as covariates.

**Results:**

Higher LC FDG‐PET signal was associated with higher meta‐temporal tau‐PET at baseline (T = 2.87, p = 0.00463) and accumulation over time (T = 2.257, p = 0.0273; Fig 2A&C). Higher LC FDG‐PET signal was associated with higher global amyloid at baseline (T = 2.066, p = 0.041) and faster amyloid accumulation over time in the dementia group (T = ‐2.74, p = 0.00904; Fig 2B&D), following a rostro‐caudal gradient.

**Conclusion:**

In a cohort with cognitive impairment, we observed steeper accumulation of Alzheimer’s disease pathology in individuals with higher LC FDG‐PET signal, with a specific amyloid‐related vulnerability for the rostral parts of the LC. Future steps will be to include cognitively unimpaired individuals to investigate the relationship between LC FDG‐PET signal and pathology accumulation in early stages of the disease process.